# Mutation analysis of *RAD51D* in non-*BRCA1/2* ovarian and breast cancer families

**DOI:** 10.1038/bjc.2012.87

**Published:** 2012-03-13

**Authors:** D J Osher, K De Leeneer, G Michils, N Hamel, E Tomiak, B Poppe, K Leunen, E Legius, A Shuen, E Smith, J Arseneau, P Tonin, G Matthijs, K Claes, M D Tischkowitz, W D Foulkes

**Affiliations:** 1 Program in Cancer Genetics, Departments of Oncology and Human Genetics, Gerald Bronfman Centre for Clinical Research in Oncology, McGill University, 546 Pine Avenue West, Montreal, Quebec H2W 1S6, Canada; 2 Department of Medical Genetics, McGill University Health Centre, Montreal, Quebec, Canada; 3 Center for Medical Genetics, Ghent University Hospital, Ghent, Belgium; 4 Center for Human Genetics, University of Leuven, Leuven, Belgium; 5 Department of Genetics, Children's Hospital of Eastern Ontario, Ottawa, Ontario, Canada; 6 Faculty of Medicine, University of Ottawa, Ottawa, Ontario, Canada; 7 Division of Gynecological Oncology, Department of Obstetrics and Gynecology, University Hospital Leuven, University of Leuven, Leuven, Belgium; 8 Department of Pathology, McGill University Health Centre, McGill University, Montreal, Quebec, Canada; 9 Department of Medicine, McGill University and The Research Institute of the McGill University Health Centre, Montreal, Quebec, Canada; 10 Lady Davis Institute, Segal Cancer Centre, Jewish General Hospital, McGill University, Montreal, Quebec, Canada

**Keywords:** double-strand DNA repair, hereditary, ovarian carcinoma, PARP inhibitors, RAD51D, RAD51C

## Abstract

**Background:**

Recent data show that mutations in *RAD51D* have an aetiological role in ovarian carcinoma, yet mutations do not appear to be associated with an increased risk for breast cancer. We studied ovarian and breast cancer families having at least one woman affected by ovarian carcinoma, to assess the importance of *RAD51D* mutations in such families.

**Methods:**

The coding region of the *RAD51D* gene was analysed in 175 *BRCA1/2*-negative families with family histories of both ovarian and breast cancer ascertained from two Canadian and two Belgian institutions.

**Results:**

We identified one previously reported deleterious mutation, p.Arg186^*^ (c.556C>T), and two novel variants; missense substitution p.Cys119Arg and an intronic variant c.83-26A>G. p.Arg186^*^ segregated with the disease in the family and two ovarian carcinomas available for analysis showed loss of the wild-type allele, but the novel variants are likely neutral.

**Conclusion:**

*RAD51D* should be included in genetic screening of ovarian cancer families that do not have *BRCA1*/*BRCA2* mutations. We show that mutations are more likely to be found in families with two or more ovarian cancers, or in probands with first-degree relatives with ovarian cancer, and we feel testing should be preferentially offered to affected women from such families.

For many years, hereditary ovarian cancer was thought to be mainly, if not entirely, attributable to mutations in the *BRCA1*/*BRCA2* breast cancer susceptibility genes (Gayther *et al*, 1999). While mutations in additional genes such as *PALB2*, *CHEK2*, *ATM*, and *BRIP1* were also found to predispose to breast cancer (Shuen and Foulkes, 2011), until recently, no other genes were found to be mutated in hereditary ovarian cancer. *RAD51C* has now emerged as an ovarian cancer susceptibility gene. Pathogenic mutations were identified in families with histories of both ovarian and breast cancer, in familial ovarian cancer alone and in those with unselected ovarian cancer. The mutation frequency observed in patients with unselected or hereditary breast cancer only was not significantly different from that observed in population controls, suggesting that mutations in *RAD51C* do not increase the risk of breast cancer *per se* (Meindl *et al*, 2010; Pelttari *et al*, 2011; Romero *et al*, 2011; Vuorela *et al*, 2011; Thompson *et al*, 2012).

Recently, another member of the *RAD51* family of paralogs, *RAD51D*, was found to be mutated in women affected by familial ovarian cancer, with or without breast cancer. Loveday *et al* (2011) found eight truncating mutations in 911 families having at least one case of ovarian cancer and one case of breast cancer. They found one truncating mutation in 1060 population controls (Loveday *et al*, 2011). Mutations were more prevalent in families with more than one ovarian cancer: four mutations were identified in 235 families (1.7%) with 2 or more ovarian cancer cases. Remarkably, 3 of these mutations were found in 59 families (5.1%) with 3 or more ovarian cancer cases. Finally, 4 mutations were identified in 676 families having only one case of ovarian cancer (0.6%). By contrast, no mutations were found in 737 individuals from families with breast cancer only. The lifetime risk of ovarian cancer for a *RAD51D* mutation carrier was estimated to be 10% by age 80.

Despite their apparent rarity, determining *RAD51D* mutation status is important for the female relatives of affected patients, as this knowledge may allow them to make informed decisions about preventive options to mitigate their elevated risk for disease. Furthermore, as *RAD51D* is involved in DNA repair through homologous recombination (HR), it is possible that carcinomas arising in patients carrying *RAD51D* mutations will be sensitive to chemotherapeutic agents that target this pathway, such as cisplatin and the poly (ADP-ribose) polymerase (PARP) inhibitor olaparib, as demonstrated in *BRCA1/2* mutation-carrier cancer patients (Banerjee *et al*, 2010; Loveday *et al*, 2011).

Here we report our analysis of *RAD51D* in 175 ovarian and breast cancer pedigrees, having at least one case of ovarian cancer in which *BRCA1* and *BRCA2* mutations were previously ruled out.

## Materials and methods

### Cases and case selection

Ovarian and breast cancer families were recruited from Cancer Genetics Clinics in Montreal, Ottawa, Ghent, and Leuven, and were eligible if at least one case of ovarian carcinoma was reported in a first, second, or third-degree relative, or if the proband was affected with ovarian cancer (Table 1A). All probands consented to participate in the study, which was approved by relevant Institutional Review Boards. In total, 175 cases from unrelated families were screened (36 from Montreal and Ottawa, 45 from Ghent, and 94 from Leuven). All patients were negative for *BRCA1* and *BRCA2* mutations based on full gene sequencing and large deletion screening, with the exception of Ashkenazi Jewish (AJ) cases (*n*=6), who were only screened for the AJ common mutation panel.

### Mutation analysis

**Figure 1:**
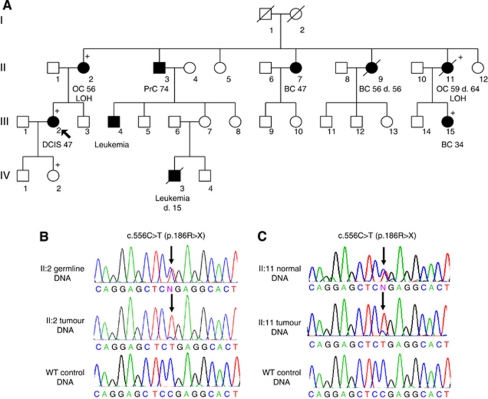
(**A**) Pedigree of family from Montreal, carrying the deleterious mutation p.Arg186^*^ (c.556C>T) in *RAD51D*. Individual II:2 had clear cell ovarian carcinoma, and II:11 had high-grade, serous ovarian carcinoma. (**B**) Sequencing results from individual II:2 showing the heterozygous mutation in germline DNA and loss of the wild-type allele in DNA extracted from the patient's ovarian tumour as compared with wild-type control DNA. Germline DNA was obtained from lymphocytes. (**C**) Sequencing results from individual II:11 showing a heterozygous mutation in normal DNA and loss of the wild-type allele in DNA extracted from the patient's ovarian tumour, compared with wild-type control DNA. Normal DNA was extracted from macro-dissected normal tissue from the patient's ovarian FFPE tumour block. Abbreviations: BC=breast cancer; DCIS=ductal carcinoma *in situ*; OC=ovarian cancer; PrC=prostate cancer.

Genomic DNA from patient leukocytes and/or saliva was extracted according to standard methods. Analysis was performed in laboratories in Ghent, Leuven, and Montreal. DNA from patients recruited in Ottawa was analysed in Montreal. Primers, PCR, and sequencing conditions used in Montreal were as described (Loveday *et al*, 2011). In Ghent and Leuven, DNA was analysed by high-resolution melting curve analysis on the 96-well Lightscanner instrument (Idaho Technology, Salt Lake City, UT, USA), followed by sequencing of the fragments with aberrant melting curves. Primer sequences are available in Supplementary Table 1A. DNA from the mutation-positive case was sequenced on both strands for verification, and DNA samples from her relatives were obtained and tested for the presence of mutation by sequencing the exon of interest (Figure 1). Detailed methods including bioinformatics mutation analysis are provided in the Supplementary Text.

### Loss of heterozygosity analysis

Ovarian and breast cancer formalin-fixed, paraffin-embedded (FFPE) tissue blocks were obtained, where possible, from affected members of the *RAD51D* mutation-positive family. Slides were cut and stained with haematoxylin and eosin, and all pathology material was reviewed by a single pathologist (JA) to confirm pathology and identify areas enriched in tumour cells. Tumour DNA was extracted from macro-dissected FFPE tissue using a Qiagen QIAamp DNA FFPE kit (QIAGEN Inc., Toronto, ON, Canada) according to the manufacturer's recommendations. To detect loss of allele heterozygosity (LOH), the genomic region containing the point mutation was amplified by PCR and sequenced. Relative peak amplitudes at the heterozygous mutation site on the chromatograms from non-tumour and tumour DNA were compared.

### cDNA analysis

The putative splice-site variant *RAD51D* c.83-26A>G was tested by splice-site prediction programmes for its potential to alter splicing. Subsequently, cDNA analysis was performed to verify the *in silico* results. Total RNA, treated with a nonsense-mediated mRNA decay inhibitor (puromycin), was extracted from short-term PHA/IL2-stimulated lymphocyte cultures of the patient and from controls not carrying the splice-site alteration. The cDNA was synthesised from 1 *μ*g of RNA with a two-step RT–PCR (iScript cDNA synthesis kit, Bio-Rad, Nazareth Eke, Belgium). Primers spanning exon 1 to exon 4 were designed (F: 5′-CCTCCTCCTCTCTCCTTTC-3′ and R: 5′-CCTACAATTTCAGTCACTTCTCCAG-3′). The PCR conditions were identical to those used for mutation analysis. Amplification products were Sanger-sequenced in both directions.

## Results

The mutation screen of 175 cases identified one deleterious nonsense mutation, p.Arg186^*^ (c.556C>T), in a Canadian family. This pathogenic mutation was previously described in two unrelated families (Loveday *et al*, 2011). We evaluated the presence of the mutation in samples from four of the proband's relatives, from whom genetic material was available; three individuals affected with ovarian or breast cancer were carriers of the family mutation, and the proband's unaffected daughter was also a carrier (Figure 1A). Pathology material was available from the two ovarian cancers, an invasive breast cancer and the proband's ductal carcinoma *in situ* (DCIS). On pathology review, one ovarian tumour was found to be a clear cell carcinoma and the other was a high-grade serous carcinoma. Both ovarian cancers showed LOH of the wild-type allele (Figure 1B and C). The diagnoses of the proband's DCIS and the cousin's invasive breast cancer were also confirmed by review of the pathology material, but available material did not allow for LOH analysis.

Three additional variants were identified (Supplementary Table 1B). The first, p.Cys119Arg (c.355T>C), is a novel missense variant observed in a Belgian family, and is predicted to be tolerated by SIFT analysis and benign by Polyphen2 analysis. The second, p.Gly265Arg (c.793G>A), also a missense identified in a Belgian family, is predicted to be damaging, based on the SIFT and Polyphen2 analyses. However, this variant did not segregate with the disease in the carrier family (data not shown) and was previously observed in one control, but not in cases (Loveday *et al*, 2011). Both these variants are most likely benign. The third variant, a novel intronic A>G substitution, was identified 26 nucleotides upstream of exon 2 (c.83-26A>G) in a Belgian family. Segregation of the variant within the family remains uncertain, as none of the proband's relatives who are affected with ovarian cancer are available for genetic testing. The proband's sister, who had breast cancer at age 58, but no ovarian cancer, tested negative for the variant (Supplementary Figure 1). Three splice-site prediction programs suggest that creation of a novel acceptor site and elimination of a branch point are likely. The cDNA analysis, however, did not reveal aberrant splicing. A novel alternative transcript containing an out-of-frame skip of exon 3 was present in the patient and all negative control samples (r.145_263del; p.Ala49SerfsX2); as it leads to a premature stop codon, this transcript is most likely not functional. These data do not confirm the *in silico* predictions and suggest that c.83-26A>G is a neutral variant.

Finally, we identified one synonymous variant and two non-synonymous variants that are reported in dbSNP to have population frequencies near to or greater than 1%: p.Ser78Ser (c.234C>T, rs9901455), p.Arg165Gln (c.494G>A, rs4796033), and p.Glu233Gly (c.698A>G, rs28363284; Supplementary Table 1B).

## Discussion

In 175 families selected, having one or more ovarian cancer cases, we identified one deleterious truncating p.Arg186^*^ mutation in *RAD51D*, previously identified in two unrelated families from the UK (Loveday *et al*, 2011). As our family of interest is of Anglo-Canadian origin, it is possible that p.Arg186^*^ is a founder mutation in the British population; however, further study will be required to confirm this hypothesis in a larger series of ethnically selected cases. This pathogenic mutation was observed once among 51 families with 2 or more cases of ovarian cancer (2.0%), and no mutations were found in 124 families with only 1 case of ovarian cancer (*P*=0.29; Table 1B). Although not statistically significant, these numbers support the findings of Loveday *et al* (2011), indicating that two cases of ovarian cancer in a family are required to provide a greater than 1% chance of identifying a mutation in *RAD51D*. Moreover, in cases where the individual with ovarian carcinoma is not available for testing, it will be important to test the person closest in relationship to an ovarian carcinoma case, irrespective of whether they are affected by cancer or not (Table 1A).

Poly (ADP-ribose) polymerase inhibitors impede single-stranded DNA repair, forcing cells to use HR to mend these breaks, and cells with a defective HR pathway will undergo apoptosis in a synthetically lethal response (Banerjee *et al*, 2010). Thus, *RAD51D* mutation carriers may benefit from PARP inhibitors as do patients with inactivating mutations in other HR genes, such as *BRCA1*, *BRCA2*, and *PALB2*. Loveday *et al* (2011) showed in tumour cells that short interfering RNAi reagents targeting *RAD51D* caused sensitivity to the PARP inhibitor olaparib, similar to the effect seen when *BRCA1* or *BRCA2* are silenced. In consequence, a patient known to be a carrier could receive PARP inhibitors early in treatment (Banerjee *et al*, 2010), making clinical genetic testing of *RAD51D* mutations in *BRCA1/2*-negative patients with one or more familial ovarian cancers in the pedigree potentially highly beneficial. Given the lifetime risk for the disease, unaffected carriers may consider early and increased screening or preventive ovarian surgery. The low yield in families having only one ovarian cancer may make gene-specific mutation testing of *RAD51D* impractical for the majority of ovarian/breast cancer families. However, as many groups move to whole-exome sequencing or to exon array panels focused on capturing variation in a large number of relevant genes, this concern will become less limiting. Exome capture and DNA sequencing of 316 high-grade serous adenocarcinomas, however, did not identify any mutations in *RAD51D*, although 20% of tumours carried a germline or somatic mutation in *BRCA1* or *BRCA2* (Cancer Genome Atlas Research Network, 2011).

Mutations in both *RAD51C* and *RAD51D* seem to primarily increase risk for ovarian cancer, and are present at greater frequency in women with breast cancer in the context of familial ovarian cancer than in women with breast cancer in the absence of a family history of ovarian cancer, such that there may be no increased risk for breast cancer when there are no reported cases of ovarian cancer in the family (Loveday *et al*, 2011; Pelttari *et al*, 2011). It is likely that the apparent excess of *RAD51C/D* mutation carriers among breast cancer cases occurring in ovarian/breast cancer pedigrees is solely due to ascertainment bias (Loveday *et al*, 2011), but it is also possible that there is a non-multiplicative interaction between rare, moderately penetrant *RAD51C/D* mutations and common, low-penetrant SNPs that slightly increase the risk for both breast and ovarian cancer.

In conclusion, we have identified one pathogenic mutation, p.Arg186^*^, in 175 probands from ovarian/breast cancer families. In the p.Arg186^*^ proband's family, two relatives were confirmed to have been diagnosed with ovarian carcinoma, and both carried the mutated allele and showed loss of the wild-type allele. Testing of *RAD51D* in women with ovarian carcinoma, who have at least one relative with ovarian carcinoma, is likely to identify mutations in 1–5% of cases. Testing in the context of this low yield may be justified in view of the opportunities for prevention and treatment that these results may provide.

## Figures and Tables

**Table 1:** Total patient population screened

**(A) By proximity to ovarian carcinoma case**					
**175 Probands from unrelated families**	**Total**	**FDR with OC**	**SDR with OC**	**TDR with OC**	**No relatives with OC**
No. of ovarian cancer probands	52	17	3	3	29
No. of breast/ovarian cancer probands	26	3	4	0	19
No. of breast cancer probands	97	63	31	3	0^a^
Total probands screened	175	83	38	6	48
*RAD51D* mutations identified	1	1	0	0	0
**(B) By all groups for *RAD51D* mutations**					
**175 Probands from unrelated families**	**Total**	**4 OC in family**	**3 OC in family**	**2 OC in family**	**1 OC in family**
No. of ovarian cancer probands	51	3	2	15	31
No. of breast/ovarian cancer probands	24	1	0	6	17
No. of breast cancer probands	100	1	1	22	76
Total probands screened	175	5	3	43	124
*RAD51D* mutations identified	1	0	0	1	0

Abbreviations: OC=ovarian cancer patient; FDR=first-degree relative; SDR=second-degree relative; TDR=third-degree relative.

a By definition.
