# Supplementary material for: Mutation analysis of RAD51D in non-BRCA1/2 ovarian and breast cancer families
Source: Br J Cancer. 2012 Mar 13;106(8):1460–3. doi: 10.1038/bjc.2012.87 (PMC3326673; doi:10.1038/bjc.2012.87)
Supplement: Supplementary Information [file bjc201287x1.doc]

Supplemental Information

Supplemental Tables

Supplemental Table 1A – Primers

| **RAD51D primers used in Montreal (as per Loveday et al.)** | | | |
| --- | --- | --- | --- |
| **Exon** | | **Forward** | **Reverse** |
| **1** | | GCCTCCTCCTCTCTCCTTTC | CACCCTTCCTGAGCCTCTC |
| **2** | | GGGTAGAATTGACACCCCATT | TGACTTCTGACTCCAAGTGACC |
| **3** | | AAAGGGAGCAGAGGGTTCTC | ATGTCCTGACCCCTTTCCTT |
| **4** | | TGGCCAGTGATGTTCAAAGA | CCCATTAGTACGCTGAAGCTC |
| **5** | | GGACTCAGCCCATTTGTGTT | AGCAAGTTTGAAGGCAAGGA |
| **6** | | CTGAGTCCTTGCATCCAGGT | ATTGCACATCTGCATTTCCA |
| **7 - 8** | | CTTGCTGTATTTGGGATGGG | TTTGGGGTTCAGAAGCTGAC |
| **9 -10** | | CTCTCCGTAAAATGAAGCGG | TAAACAGCAGGCGTTACTGG |
| **RAD51D primers used in both Ghent and Leuven** | | | |
| **Exon** | **Forward** | | **Reverse** |
| **1** | M13-GCCTCCTCCTCTCTCCTTTC | | M13-AAGCGCCCTGCAGGTATGC |
| **2** | M13-GGAAAAACCAAGTGTGGGAGG | | M13-CCAGCTGGCTTGGGATGG |
| **3** | M13-GGGTGAATGACACCCTGGG | | M13-GCTGAGAGGAGGCCCCAT |
| **4** | M13-TGTCTTACTTTTCTTCTTGGTACCTT | | M13-TGGGCTATGCATCTACCAC |
| **5** | M13-GAGCTCCCATCTGGACC | | M13-CTTACAATGTTAAGGGATAATGGG |
| **6** | M13-CAGCCTTACCTTGACCC | | M13-CCCACAGAGATAGCACCTAGA |
| **7** | M13-GCTTGTCCTGTAATGTGTCCTA | | M13-GCCAGAGACCAGACTCC |
| **8** | M13-GCTCTGGAGTCTGGTCT | | M13-GAAGCTGACATTTAAGGGAAATAAAG |
| **9** | M13-CGGTAGTAATCGATGTCCTCT | | M13-GGTATGGAAACCACCCTCC |
| **10** | M13-CAACTAAAGAAGCTACCCACTTG | | M13-GTAAACAGCAGGCGTTACT |
|  | Forward M13 (used in Ghent): CACGACGTTGTAAAACGAC | | |
|  | Forward M13 (used in Leuven): TGTAAAACGACGGCCAGT | | |

Supplemental Table 1B – Sequences variants detected at all sites (Montreal, Ghent, Leuven)

| **Sequence variants** | |  |  |  |  |  |  |
| --- | --- | --- | --- | --- | --- | --- | --- |
| **Mutation** | **Protein Change** | **dbSNP** | **Freq** | **In silico analyses** | | | **Previously identified [result]** |
|  |  |  | *(alleles n= 350)* | *SIFT* | *Polyphen* | *Splice site* |  |
| **c.234 C>T** | p.Ser78Ser | rs9901455 | 0.10 (36/350) | - | - | Benign | Loved*ay et a*l., 2011 [non-pathogenic] |
| **c.494G>A** | p.Arg165Gln | rs4796033 | 0.12 (41/350) | Tolerated | Benign | Benign | Loved*ay et a*l., 2011 [non-pathogenic] |
| **c.698A>G** | p.Glu233Gly | rs28363284 | 0.02 (7/350) | Tolerated | Probably damaging | Benign | Loved*ay et a*l., 2011; Rodriguez-Lop*ez et a*l., 2004 [non-pathogenic] |
| **c.904-3C>T** | - | - | 0.003 (1/350) | - | - | Benign | Loved*ay et a*l., 2011 [non-pathogenic] |
| **c.793G>A** | p.Gly265Arg | - | 0.003 (1/350) | Affects Protein function | Probably damaging | Benign | Loved*ay et a*l., 2011 [non-pathogenic] |
| **c.83-26A>G** | - | - | 0.003 (1/350) | - | - | Possible creation of novel acceptor site (*) and abolishment of branch point | - |
| **c.355T>C** | p.Cys119Arg | - | 0.003 (1/350) | Tolerated | Benign | Benign | - |
| **c.556C>T** | p.Arg186Stop | - | 0.003 (1/350) | - | - | - | Loved*ay et a*l., 2011 [pathogenic] |

(*) predicted by SpliceSiteFinder-like, MaxentScan, Human Splicing Finder. In silico predictions, however were not confirmed by RT-PCR and cDNA sequencing.

Supplemental Text

**Expanded Materials and Methods**

*Mutation Analysis*

In Ghent and Leuven samples, PCR was carried out in 15µL reaction volumes. The amplification mixture consisted of 1.5 mM MgCl_2_ (Invitrogen), 1x PCR buffer (Invitrogen), 3% DMSO (VWR International), 0.2 µM of both forward and reverse primers, 200 µM of each dNTP, 0.05 U Platinum® Taq DNA polymerase (Invitrogen), 1x LCGreen Plus, and approximately 50 ng DNA. All 10 amplicons were amplified by PCR with an initial denaturation step at 95°C for 5 min, followed by 35 cycles of denaturation at 95°C for 30 s, annealing at 58°C for 30 s, and extension at 72°C for 30s. Final extension was accomplished at 72°C over 7 min. Melting acquisition was performed on the 96-well Lightscanner (Idaho Technology Inc.). 10 µL of PCR product was transferred to 96-well plates suitable for high-resolution melting analysis (4Titude plates (BioKé)). To prevent evaporation during heating, PCR products were covered with a mineral oil overlay. The plates were heated from 70°C to 98°C at 0.1°C/s. Melting curve analysis was performed with Lightscanner software (version 2.0). Shape differences were further analyzed by subtracting the curves from a reference curve, generating a difference plot, where fluorescence of all curves is plotted versus temperature. Aberrant melting curves were sequenced using the BigDye Terminator v3.1 cycle sequencing kit (Applied Biosystems, Foster City, CA). The samples were analysed on the ABI 3730 DNA Sequencer (Applied Biosystems). In Ghent the resulting sequence data was analyzed with Seqscape software, version 2.5 (Applied Biosystems), and in Leuven SeqPilot software, version 3.5.0 (JSI) was used. In the Montreal *RAD51D* patient cohort, PCR was carried out in 50µL reaction volumes. The amplification mixture consisted of 1mM MgCl_2_, 5ul of 10x PCR Buffer, 0.4µM of each forward and reverse primers, 200 mM of each dNTP (Qiagen Missisauga Ontario, Canada), 0.8 U of HotStar Plus Taq DNA polymerase, and appproximately 100ng DNA. All 10 amplicons were amplified byPCR with an initial denaturation step at 95°C for 15 mins, and followed by 35 cycle of denaturation at 95°C for 30 s, annealing at 60°C for 30 s, and extension at 72°C for 30 s. Final extension was accomplished at 72°C for 3 min. *RAD51D* PCR products were were checked by agarose gel electrophoresis, then purified and sequenced. Sequence data were analyzed by using the Lasergene software from DNASTAR (Madison, Wisconsin). Human Genome Variation Nomenclature was used to name sequence variants with Ensembl transcript ENST00000345365 as a reference sequence. In the *RAD51D* deleterious mutation positive proband, the PCR product was sequenced in the forward and reverse directions for confirmation. Where possible, DNA samples from the *RAD51D* mutation positive family were obtained and tested for the familial mutation by sequencing the exon of interest. Disease-causing potential of identified mutations was assessed using the following publicly available programs:

SIFT (<http://sift.jcvi.org/>),

MaxentScan (<http://genes.mit.edu/burgelab/maxent/Xmaxentscan_scoreseq.html>),

Human Splicing Finder (<http://www.umd.be/HSF/>),

Alamut (<http://www.interactive-biosoftware.com/alamut/doc/2.0/splicing.html>).

Supplemental Figure

Supplemental Figure 1

**
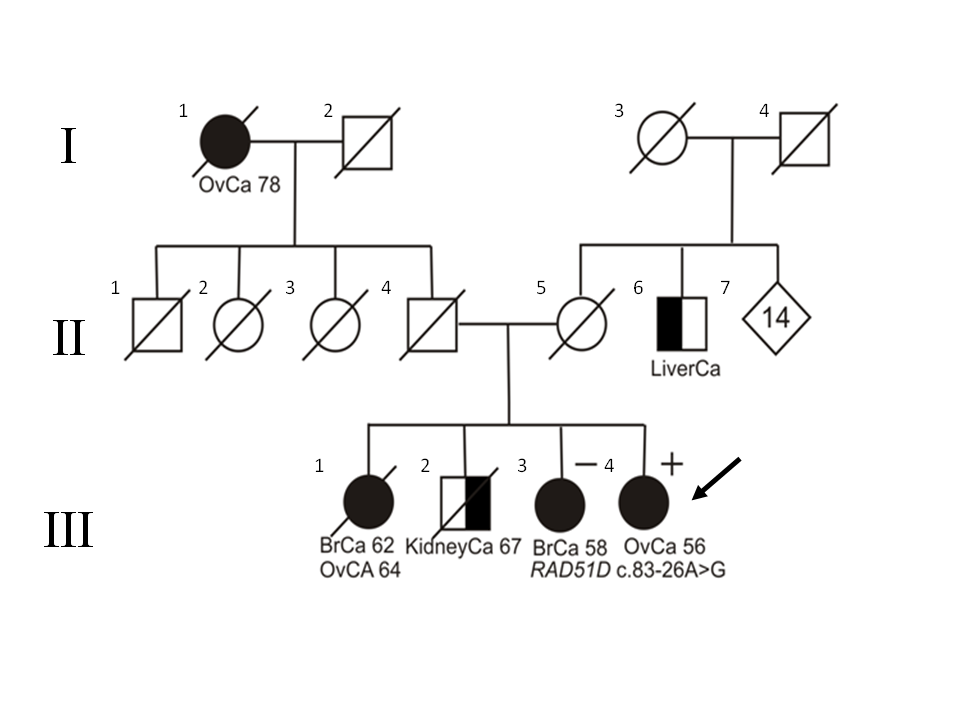
**

Pedigree of family from Ghent patient cohort carrying the *RAD51D* c.83-26A>G variant. Individual III:3 tested negative for the familial *RAD51D* variant. BrCa, breast cancer; OvCa, ovarian cancer. Arrow indicates the proband.

Supplemental Reference List

Loveday C, Turnbull C, Ramsay E, Hughes D, Ruark E, Frankum JR, Bowden G, Kalmyrzaev B, Warren-Perry M, Snape K, Adlard JW, Barwell J, Berg J, Brady AF, Brewer C, Brice G, Chapman C, Cook J, Davidson R, Donaldson A, Douglas F, Greenhalgh L, Henderson A, Izatt L, Kumar A, Lalloo F, Miedzybrodzka Z, Morrison PJ, Paterson J, Porteous M, Rogers MT, Shanley S, Walker L, Eccles D, Evans DG, Renwick A, Seal S, Lord CJ, Ashworth A, Reis-Filho JS, Antoniou AC, Rahman N (2011) Germline mutations in RAD51D confer susceptibility to ovarian cancer. *Nat Genet* **43** (9): 879-882, doi:ng.893 [pii];10.1038/ng.893 [doi]

Rodriguez-Lopez R, Osorio A, Ribas G, Pollan M, Sanchez-Pulido L, de la Hoya M, Ruibal A, Zamora P, Arias JI, Salazar R, Vega A, Martinez JI, Esteban-Cardenosa E, Alonso C, Leton R, Urioste AM, Miner C, Armengod ME, Carracedo A, Gonzalez-Sarmiento R, Caldes T, Diez O, Benitez J (2004) The variant E233G of the RAD51D gene could be a low-penetrance allele in high-risk breast cancer families without BRCA1/2 mutations. *Int J Cancer* **110** (6): 845-849, doi:10.1002/ijc.20169 [doi]
